# Contribution of the CK2 Catalytic Isoforms α and α’ to the Glycolytic Phenotype of Tumor Cells

**DOI:** 10.3390/cells10010181

**Published:** 2021-01-18

**Authors:** Francesca Zonta, Christian Borgo, Camila Paz Quezada Meza, Ionica Masgras, Andrea Rasola, Mauro Salvi, Lorenzo A. Pinna, Maria Ruzzene

**Affiliations:** 1 Department of Biomedical Sciences, University of Padova, 35131 Padova, Italy; francesca.zonta@unipd.it (F.Z.); christian.borgo@unipd.it (C.B.); camilapaz.quezadameza@studenti.unipd.it (C.P.Q.M.); andrea.rasola@unipd.it (A.R.); mauro.salvi@unipd.it (M.S.); lorenzo.pinna@unipd.it (L.A.P.); 2 CNR Neuroscience Institute, 35131 Padova, Italy; ionica.masgras@unipd.it

**Keywords:** CK2, casein kinase 2, isoforms, cancer metabolism

## Abstract

CK2 is a Ser/Thr protein kinase overexpressed in many cancers. It is usually present in cells as a tetrameric enzyme, composed of two catalytic (α or α’) and two regulatory (β) subunits, but it is active also in its monomeric form, and the specific role of the different isoforms is largely unknown. CK2 phosphorylates several substrates related to the uncontrolled proliferation, motility, and survival of cancer cells. As a consequence, tumor cells are addicted to CK2, relying on its activity more than healthy cells for their life, and exploiting it for developing multiple oncological hallmarks. However, little is known about CK2 contribution to the metabolic rewiring of cancer cells. With this study we aimed at shedding some light on it, especially focusing on the CK2 role in the glycolytic onco-phenotype. By analyzing neuroblastoma and osteosarcoma cell lines depleted of either one (α) or the other (α’) CK2 catalytic subunit, we also aimed at disclosing possible pro-tumor functions which are specific of a CK2 isoform. Our results suggest that both CK2 α and α’ contribute to cell proliferation, survival and tumorigenicity. The analyzed metabolic features disclosed a role of CK2 in tumor metabolism, and suggest prominent functions for CK2 α isoform. Results were also confirmed by CK2 pharmacological inhibition. Overall, our study provides new information on the mechanism of cancer cells addiction to CK2 and on its isoform-specific functions, with fundamental implications for improving future therapeutic strategies based on CK2 targeting.

## 1. Introduction

The rewiring of metabolism is a hallmark of cancer, and the shift in ATP generation from oxidative phosphorylation to glycolysis and the production of lactate even under oxygen-rich conditions (aerobic glycolysis) is a major metabolic feature of many cancer cells [1].

CK2 (previously known as casein kinase 2) is a Ser/Thr protein kinase, ubiquitously expressed, but particularly abundant in cancer cells [2,3,4]. It phosphorylates a huge number of proteins, and is involved in several cellular processes, with a major role in supporting cell proliferation and survival [5,6]. It is usually present in cells as a constitutively active tetrameric enzyme, composed of two catalytic (α or α’) and two regulatory (β) subunits [7]. Catalytic subunits are active even in the absence of the β subunit, but the relevance of the monomeric form in cells has not been proved, yet. However, several evidences support a possible pro-tumor function of the isolated catalytic subunits [8,9]. The two isoforms α and α’ are very similar in structure and often considered overlapping in functions; however, also isoform-specific functions have been described [10,11] and a possible differential role in tumors has been hypothesized [12]. CK2 involvement in cancer has been known since many years, and has been described as a “lateral action”, where, by phosphorylating crucial substrates, it potentiates signaling related to most cancer hallmarks. However, CK2 importance in cancer metabolism is less documented, even though its functions in hormonal regulation of carbohydrate metabolism have been described [13]. Zhang and colleagues [14] observed that glucose uptake and lactate secretion were lower in bladder cancer cell lines where CK2α expression was knocked down. Consistently, they found that a number of glycolysis-related genes were less expressed in these cells, or in w.t. bladder cancer cells treated with the CK2 inhibitor CX-4945, and they interpreted this as a consequence of the CK2-dependent reduction of S473 Akt phosphorylation. This paper only refers to CK2α isoform. Yang et al. [15] reported about the modulation of pyruvate kinase M isoforms by CK2. They found that, in colon cancer cells overexpressing CK2α, the constitutively active PKM1 was downregulated through a proteasome-dependent mechanism, while the level of PKM2 (whose induction contributes to the Warburg effect) was unchanged, but it was more localized in the nucleus, where it promotes a higher expression of LDHA.

Another study [16], by an isotope tracer metabolite analysis, showed that glucose is more important than glutamine in sustaining the tumor phenotype of CK2α-overexpressing cells, where ROS levels were found to decrease.

The CK2 implication in cancer energetics can be also inferred by its regulatory role on Akt and its effectors mTORC1 and β-catenin [17,18], ultimately affecting the modulation of key metabolic targets, as recently described [19]. Moreover, CK2 plays a role in adipocyte metabolism [20], and its possible implication in cancer aberrant lipid metabolism has been hypothesized [21].

These observations prompted us to investigate more in depth how metabolic features of tumor cells depend on CK2, especially focusing on the specific functions of the individual catalytic isoforms. To this aim, we exploited the CRISPR-Cas9 technology to turn off the expression of either α or α’ CK2 catalytic subunits in SK-N-BE (human neuroblastoma) and U2OS (human osteosarcoma) cells, giving rise to clones hereinafter denoted as KOα and KOα’, and we analyzed how this impacted on cellular and metabolic features.

## 2. Materials and Methods

### 2.1. Antibodies and Chemicals

Anti-CK2α/α’ (MCA3031Z) was purchased from Bio-Rad (Hercules, CA, USA), anti-CK2 β (ab76025) and anti-pSer129 Akt (ab133458) from Abcam, anti-total Akt (clone H-136), anti-Hk2 (clone 1A7) and anti-total LDH (clone H-160) from Santa Cruz Biotechnologies (Dallas, TX, USA), anti-β actin (A2228) and anti-tubulin (T5168) from Sigma; anti-Hif-1α (127309) from GeneTex.

The CK2 inhibitor CX-4945 (5-[(3-Chlorophenyl)amino]-benzo[c]-2,6-naphthyridine-8-carboxylic) was purchased from MedChemExpress and the chemical inducer of hypoxia DMOG (dimethyloxallyl glycine) was from Sigma. Inhibitors solutions at different concentrations were prepared in 100% DMSO (Sigma, St. Louis, MO, USA).

### 2.2. Cell Culture

For this study, we used the human neuroblastoma cell line SK-N-BE (synonyms SK-N-BE(2)), and the human osteosarcoma cell line U2OS.

All cells (wild type, w.t. and KO clones, see below) were grown in Dulbecco’s Modified Eagle Medium (D-MEM, Sigma) containing 5 mM glucose, supplemented with 10% (*v/v*) fetal bovine serum (FBS) and PSG (1% penicillin and streptomycin and 2 mM L-Glutamine) (Sigma). Cells were maintained at 37 °C in a humidified atmosphere of 95% air and 5% CO_2_.

### 2.3. Generation of CK2-Knockout Cells by CRISPR-Cas9 Editing Tool

All-in-one plasmids expressing Cas9–Dasher green fluorescent protein (GFP) and the single guide RNA (sgRNA) guide (CMV-Cas9-2A-GFP, Cas9-ElecD) to target the specific CK2 subunits were purchased from ATUM (Newark, CA, USA). The sgRNA guide sequences targeting CK2 in SK-N-BE are: 5′-CCTGGATTATTGTCACAGCA-3′ (clone 1) and 5’-TTACATGTATGAGATTCTGA-3’ (clone 2) for CK2α (*Csnk2a1*), 5′-GGGTCTACGCCGAGGTGAAC-3′ (clone 1) and TTAATATCACCAACAATGAG (clone 2) (clone 2) for CK2α’ (*Csnk2a2*). The sgRNA guide sequences targeting CK2 in U2OS are: 5′-CCTGGATTATTGTCACAGCA-3′ (clone 1) and 5’-TTACATGTATGAGATTCTGA-3’ (clone 2) for CK2α (*Csnk2a1*), 5′-GGGTCTACGCCGAGGTGAAC-3′ (clone 1) and 5′-AACTGGTTCGAAAACTTGGT-3′ (clone 2) for CK2α’ (*Csnk2a2*).

Individual CK2 subunit knockout was performed as previously detailed [22]. Briefly, cells were co-transfected with 1 μg of sgRNA plasmid and Lipofectamine 3000 (Thermo Fisher Scientific, Waltham, MA, USA) according to the manufacturer’s instructions. At 48 h post-transfection GFP-positive cells were sorted in 96-well plates using fluorescence-activated cell sorting with a FACSAria II Cell Sorter (BD BioSciences, Franklin Lakes, NJ, USA). Sorted cells were expanded to obtain individual clones, which were selected by immunoblotting in order to verify the complete absence of target protein expression.

As a control, we also produced a monoclonal w.t. line, whose phenotype largely overlapped that of the w.t. cells used throughout the study. To this purpose, cells have been transfected with an GFP vector and GFP-positive cells were sorted in 96-well plates by FACS and expanded to obtain individual clones.

### 2.4. Cell Treatments and Lysis

Cells were cultured in dishes up to 70–80% confluence, then treated at the indicated time and concentration conditions with the chemical compounds (CX-4945 or DMOG), or with solvent in the case of control cells. Non-toxic concentrations were applied, ensuring viability level was >70%, as assessed by the MTT (3-(4,5-dimethylthiazol-2-yl)-3,5-diphenyltriazolium bromide) method [23]. At different time points, cells were harvested, centrifuged and washed with PBS. Cells were lysed with a buffer containing 0.5% (*v/v*) Triton X-100, as elsewhere described [24] and subjected to the Bradford quantification assay before SDS-PAGE separation and western blot analysis. 

### 2.5. Western Blotting Analysis and Titration of the CK2 Antibody Reactivity

Equal amounts of proteins (10–20 μg) from cell lysates were resolved by 7.5%, 11%, or 15% SDS-PAGE and transferred to Immobilon-P membranes (Merck-Millipore, Burlington, MA, USA) using the Lightning Blotter Transfer System (Perkin Elmer, Waltham, MA, USA). Dried membranes were then washed in TTBS buffer (50 mM Tris-HCl pH 7.5, 50 mM NaCl, 0.1% Tween-20), and incubated with the indicated antibodies. Membranes were then incubated with secondary HRP-conjugated antibody (Perkin Elmer) for 30 min and bands were detected by a chemiluminescence solution composed by 50 mM Tris, pH 9.35, 0.02% (*v*/*v*) H_2_O_2_ and 50% (*v*/*v*) luminol solution plus 30% (*w/v*) BSA, with an enhanced chemiluminescent detection system (Thermo Fisher Scientific). Immunostained bands were quantified using a 4000 mm Pro Kodak Image Station, followed by analysis with Carestream Molecular Imaging software. β-actin or α-tubulin were used as a loading control.

For the titration of the CK2 antibody reactivity, recombinant human CK2 subunits were analyzed. They were produced, purified and kindly donated by Stefania Sarno, Padova [25]. Purified proteins, dialyzed against 25 mM Tris-HCl pH 7.5 and 50% glycerol, were stored at −20 °C, before loading on SDS-PAGE. Gels were either stained by Colloidal Coomassie Blue or transferred to Immobilon-P membranes and analyzed by Western blotting with the anti-CK2α/α’ antibody.

### 2.6. Cell Growth Assay

The growth of viable cells was monitored by a Trypan blue exclusion assay. SK-N-BE (5 × 10^4^) or U2OS (2 × 10^3^) cells were seeded in triplicate in 6-well/plates. Cells were allowed to attach for 5 h, then t = 0 was considered, and the number of viable cells was monitored every 24 h up to 96 h. To this purpose, cells were washed, treated with 0.05% trypsin solution, centrifuged, resuspended in a 0.13% Trypan blue solution and counted in an hematocytometer, excluding stained (dead) cells. Unstained (viable) cells were counted in triplicate for each time point.

### 2.7. Spheroid Formation Assay

Cells were seeded in quadruplicates at 1 × 10^3^ cells/well in 200 μL of culture medium supplemented with 5% (*v*/*v*) FBS, in a 96-well plate with round bottom wells precoated with 50 μL of 1% agar in culture medium [26]. Images were taken every 24 h for 7–8 days, by using a Leica DMI4000 automated inverted microscope equipped with a Leica DFC300 FX camera (2.5× objective, magnification-changer 1.6).

### 2.8. Clonogenic Survival Assays

Cells were plated at 250 (SK-N-BE) or 100 (U2OS) cells/well in 6-well plates, in full growth medium. Media were replaced by fresh culture medium every 4 days. After 15 days, cells were washed with PBS, fixed with methanol and stained with 0.5% crystal violet. Digital images of cell colonies were obtained using a scanning device (Epson Perfection V700 Photo). Quantification of the clonogenic assay was performed with the ImageJ plugin “ColonyArea” [27] to determine the area covered by colonies in the well and the colony intensity percentage.

### 2.9. Wound-Healing Assays

Wound-healing assays were performed by creating identical wound areas into the cell monolayer using Ibidi culture-inserts (Ibidi GmbH, Cat. no. 80209). Cells were seeded in triplicate in complete culture medium on each side of the Ibidi culture-insert, into a 24-well plate, at proper cell densities (1.5 × 10^6^ SK-N-BE cells/mL and 5.5 × 10^4^ U2OS cells/mL). After 24 h, cells reached confluence, and the culture-inserts were removed in order to form a cell-free gap into the cell monolayer. Each well was washed once with PBS to remove cell debris and immediately refilled with fresh D-MEM medium supplemented with 5% (*v*/*v*) FBS and 2mM L-glutamine. The wound images were captured at this time (t = 0) and at the indicated time points (24–48 h for SK-N-BE, 2–4–8 h for U2OS) using a Leica DMI4000 automated inverted microscope equipped with a Leica DFC300 FX camera (2.5× objective, magnification-changer 1.6). Images of in vitro scratch wound healing assays were analyzed with a plugin of ImageJ software (1.52t version) adapted from [28] and relative cell migration of each cell line was measured.

### 2.10. Extracellular Lactate Concentration Analysis

Extracellular lactate concentration was measured using an L-Lactate Assay Kit (Promega, Madison, WI, USA), following the manufacturer’s instructions. Briefly, cells were seeded in a 96-well plate at the proper cell density (2 × 10^4^ SK-N-BE or 8 × 10^3^ U2OS w.t. and KO cells) in D-MEM supplemented with 1% (*v*/*v*) dialyzed FBS (Thermo Fischer Scientific); medium was collected at various time point and stored at −20 °C until analysis. The lactate concentration in each sample was detected by an enzymatic assay, which results in a luminescent product, proportional to the lactate present. Signals were detected with a luminometer plate reader (Tecan M200 Infinite Pro Microplate Reader) and expressed as average relative light units. A standard curve was built with known amounts of lactate, to identify the linear range; samples of cell culture medium were diluted 1 to 20 folds, to ensure reading within the linear range.

### 2.11. Seahorse Glycolysis Stress Test

Cellular glycolytic phenotype was analyzed by the extracellular flux analyzer Seahorse (Agilent, Santa Clara, CA, USA) (XF Analyzer for SK-N-BE cells, or XFe Analyzer for U2OS cells), performing glycolytic stress tests and following the extracellular acidification rate (ECAR) in real time, as previously reported [29]. Briefly, cells were seeded in a Seahorse 24-well assay plate in D-MEM, with 10% (*v*/*v*) FBS, 5 mM glucose and 2 mM L-Glutamine, at cell densities determined in order to have 80% confluence after 24 h (7 × 10^4^ SK-N-BE cells/well, and 2 × 10^4^ U2OS cells/well). After 24 h, cells were washed and medium was replaced with prewarmed running medium (D-MEM without phenol red supplemented with 1 mM sodium pyruvate, 30 mM sodium chloride and 2 mM L-Glutamine, pH 7.4, and incubated in a non-CO_2_ incubator at 37 °C for 60 min. ECAR were recorded in basal conditions (in the absence of glucose) and following sequential injections of 10 mM Glucose, 1 μg/mL oligomycin (mitochondrial ATP synthase inhibitor; this concentration completely abolished ATP synthase activity, as judged by the evaluation of the Oxygen Consumption Rate (OCR), not shown), 100 mM 2-DG (2-deoxyglucose, hexokinase 2 inhibitor). Glucose addition induces the glycolytic pathway, thus highlighting the “glycolytic response”. The subsequent injection of oligomycin, which blocks the mitochondrial ATP production, allows to estimate the maximal glycolysis. Lastly, administration of 2-DG measures the non-glycolytic ECAR. The “glycolytic capacity” and the “glycolytic reserve” were obtained by subtracting to the maximal glycolytic rate the residual non-glycolytic ECAR and the glycolytic response, respectively. Cells were lysed post-measurement and protein content, measured by the Bradford method, was used as a normalization value. Quantification of the different glycolytic parameters was performed with different Agilent software. XF software, which is associated to the XF analyzer and was used for SK-N-BE cells (Figure 8A,C), calculates the AUC (Area Under Curves) for each phase. In the case of SK-N-BE treatments with CX-4945 (Figure 8C), since the length of the phases was different, three points were chosen for each phase (points 1–3 for basal glycolysis; 5–7 for the response to glucose addition; 13–15 for the maximal glycolysis; 18–20 for non-glycolytic acidification). The software Wave, associated to XFe Analyzer which was used for U2OS cells (Figure 8B), for each phase considers the last point before further addition (point 3 for basal glycolysis; 6 for the response to glucose addition; 9 for the maximal glycolysis; 11 for non-glycolytic acidification), thus reports data as mpH/min.

### 2.12. Cell Viability Assay

Cell viability was detected by means of MTT reagent: cells (10^5^ cells/100 μL) were incubated for 24 h or 48 h in a 96-well plate in D-MEM supplemented with 1% (*v*/*v*) FBS, in the presence of the indicated compounds, or of DMSO vehicle, (1%, *v/v*) for control cells. 1 h before the end of the incubation, 10 μL of MTT solution (5 mg/mL in PBS) were added to each well. Incubations were stopped by addition of 20 μL of lysis solution at pH 4.7, as described elsewhere [23]. Plates were read for OD at λ590 nm, in a Titertek Multiskan Plus plate reader (Flow Laboratories, Puteaux, France). IC_50_ (concentrations inducing 50% of cell death) values were calculated with Prism 7 software (GraphPad Software, San Diego, CA, USA).

For the combined treatments, the CalcuSyn (version 2.11; Biosoft, Cambridge, GB) median effect model was used to calculate the combination index values (CI), where synergy, additivity, and antagonism are defined by CI < 1, CI = 1 or CI > 1, respectively, according to the Chou-Talalay method [30].

### 2.13. Statistical Analysis

The number of experiments is indicated in each figure legend. Quantification refers to experiments with the different KO clones of each subunit in a certain cell lines, unless differently specified. Statistical significance was evaluated by One-way Anova analysis using GraphPad Prism 7 program, setting (*) *p* < 0.05, (**) *p* < 0.01, (***) *p* < 0.001, (****) *p* < 0.0001.

## 3. Results

### 3.1. CK2 Profile in w.t. Cell Lines and KO Clones

For our study, we used SK-N-BE (neuroblastoma) and U2OS (osteosarcoma) tumor cells. For both lines, different KO clones of each subunit were obtained throughout this investigation (see Section 2), and they were variably used for performing experiments. Unless differently specified, the results shown for a certain KO clone were reproducible with the other clone of the same subunit (while, for quantifications, all data from experiments with different clones were considered). In some analysis, the two KO clones of the same subunit displayed different behaviors (suggesting possible effects due to compensating events, and/or not directly ascribable to the CK2 subunit deletion); in those cases, the results obtained with both clones are shown. 

**Figure 1:**
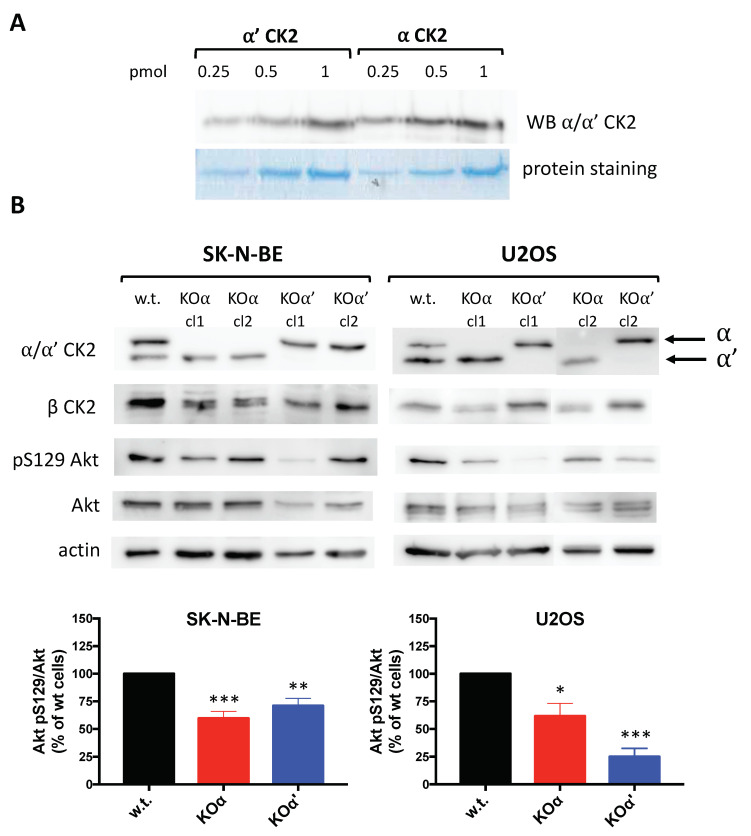
CK2 expression and activity in SK-N-BE and U2OS cells. (**A**) Titration of the antibody reactivity towards CK2 catalytic subunits. The indicated amounts of recombinant CK2 catalytic subunits (myc-α’ or α) were loaded on SDS-PAGE, and either blotted for the WB (western blot) analysis or stained by Colloidal Coomassie Blue; (**B**) CK2 expression and activity in w.t. and KO clones of the cells used for this study. 10 μg proteins from cell lysates were analyzed by WB with the indicated antibodies. The last two right lanes belong to an independent experiment. As a reporter of CK2 endogenous activity, pS129 Akt signal has been quantified, normalized to total Akt signal, and reported in the bar graph as % of w.t. cells. At least three independent experiments were performed; representative western blots are shown, while quantification in the bar graphs reports the mean values ± SEM of all experiments and of the two clones of the same KO. Statistical significance refers to w.t. cells. (*) *p* < 0.05, (**) *p* < 0.01, (***) *p* < 0.001

SK-N-BE and U2OS cells express significant amount of both α and α’. The relative percentage of the catalytic isoforms is around 70% α and 30% α’ in SKNBE, and 45% α and 55% α’ in U2OS, as assessed by using an antibody which recognizes with the same efficacy the two subunits (Figure 1A). Therefore, these two lines provide a model for two different conditions as far as the most abundant isoform is concerned. Applying the CRISPR-Cas9 technology, we produced cells of both lines that do not express either α (KOα) or α’ (KOα’) (Figure 1B). The expression of the regulatory β subunit of CK2 was roughly unchanged in KOα’ clones, while it was reduced in KOα clones, as already observed in other cell lines [31]. The CK2 cellular activity, measured through the level of the endogenous substrate pSer129 Akt [17], was reduced in the KO clones compared to w.t. cells, and the effect was more evident in KOα for SKNBE, and in KOα’ in U2OS, consistent with the proportion of the catalytic isoform suppressed (Figure 1B). 

### 3.2. Deletion of an Individual CK2 Catalytic Subunit Reduces 3D Growth, Clonogenic Potential, and Motility

First we wanted to assess if the knockout of CK2 α or α’ affects the proliferation rate of SK-N-BE and U2OS tumor cells grown in monolayer.

**Figure 2:**
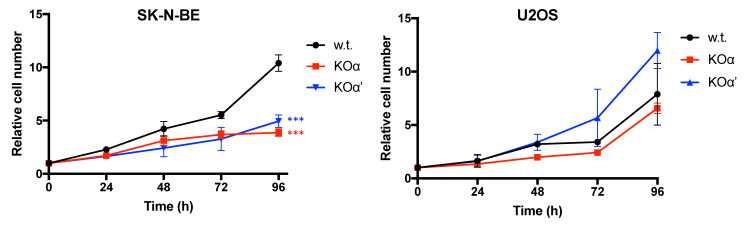
Proliferation curves of w.t. and KO clones of SK-N-BE and U2OS cells. The cell number was evaluated every 24 h, by means of Trypan blue exclusion assay (see methods for details). Experiments were performed in triplicates; at least three independent experiments were performed. The curve for each KO reports the means values ± SEM obtained with the different clones of the same KO. Significance calculated at 96 h refers to w.t. cells. (***) *p* < 0.001

In SK-N-BE neuroblastoma cells, both α and α’ KO displayed a slower proliferation compared to w.t. cells (Figure 2). Results were less clear for the U2OS osteosarcoma model: the U2OS KOα’ cells were more proliferative than the w.t. cells, while KOα were quite similar to w.t. (Figure 2).

**Figure 3:**
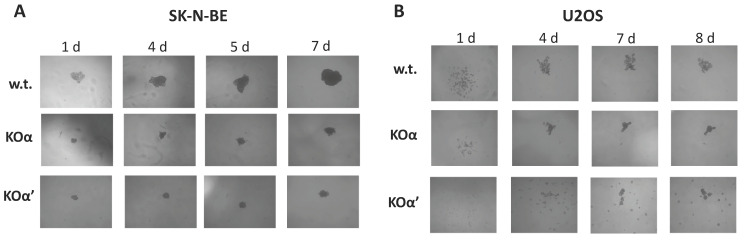
Spheroid formation potential of w.t. and KO clones of (**A**) SK-N-BE cells, and (**B**) U2OS cells were seeded in a 96-well plate precoated with 50 μL of 1% agar (see Section 2). Inverted microscope images taken at the indicated times (d = days) are shown. At least two separate experiments were performed, in quadruplicates. Representative images (SK-N-BE KOα clone 1, KOα’ clone 1; U2OS KOα clone 2, KOα’ clone 1) are shown.

To better understand the proliferative phenotypes, we decided to perform spheroid formation assays, since the growth in soft agar more closely recapitulates the actual growth of a tumor mass. We found that the 3D growth of SK-N-BE KO for either α or α’ was reduced compared to w.t. (Figure 3A), while it was quite similar for w.t. and KO clones of U2OS cells (Figure 3B).

**Figure 4:**
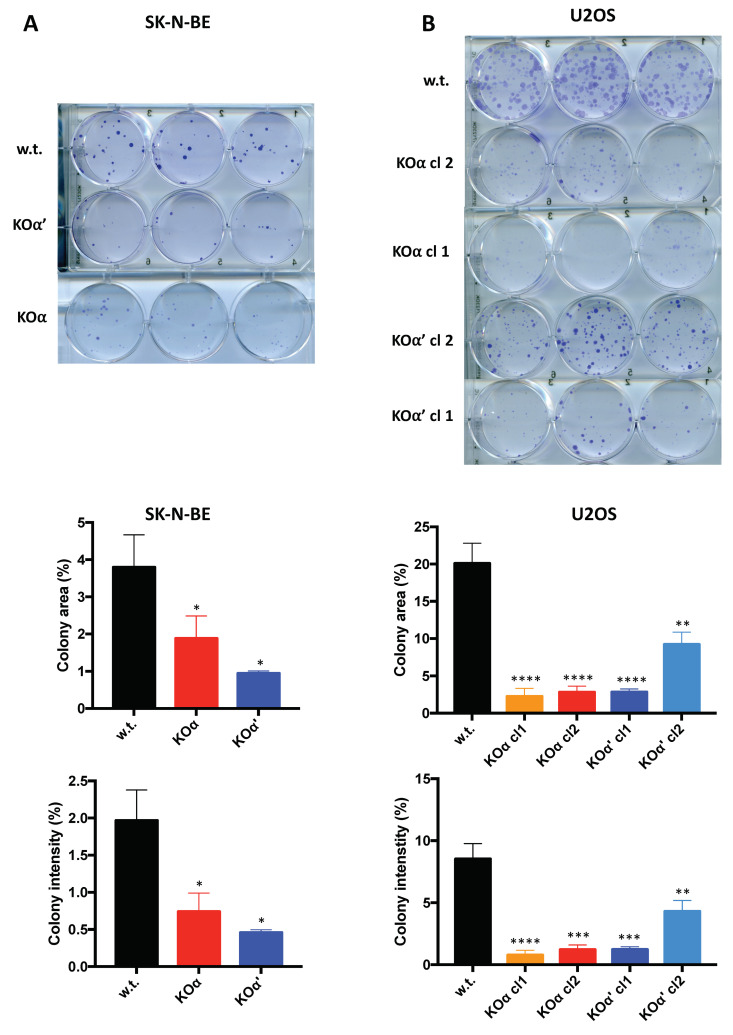
Clonogenic potential of w.t. and KO clones. (**A**) 250 SK-N-BE cells/well (w.t. or KOα clone 1, or KOα’ clone 1), or (**B**) 100 U2OS cells/well (w.t. or clones as indicated) were plated, then grown for 15 days. Colonies were stained with Crystal violet, and images were taken by a scanner device at day 15. At least two separate experiments in triplicate were performed; three representative images are shown for each clone. The bar graphs on the bottom show the quantification of the area covered by colonies (upper graph) and the colony intensity percentage (lower graph), which is related to the number of cells in a colony (mean values ± SEM). Significance refers to w.t. cells. (*) *p* < 0.05, (**) *p* < 0.01, (***) *p* < 0.001, (****) *p* < 0.0001.

To further investigate the importance of the CK2 subunits on the growth potential of tumor cells, we performed clonogenic assays; this approach provides more information, assuming that the number of colonies measures the cell survival, while the colony diameter mainly depends on the proliferation rate [32]. The clonogenic potential of both α and α’ KO of SK-N-BE was lower than that of w.t. cells (Figure 4A). Similarly, the KOα U2OS cells displayed a much lower number of colonies and smaller colonies compared to w.t. cells (Figure 4B). The clonogenic potential of two different KOα’ U2OS clones was instead quite different, although for both of them the colony size and number were reduced compared to w.t. cells (Figure 4B). Additionally, considering the higher proliferating rate of U2OS KOα’ cells (Figure 2), for the time being we cannot conclude that in these cells α’ strongly contribute to the proliferation and survival, which are instead profoundly hampered by the deletion of α.

In conclusion, our experiments highlight a lower proliferation and survival potential of all the SK-N-BE KO clones and of the U2OS KOα clones, compared to their w.t. counterpart.

**Figure 5:**
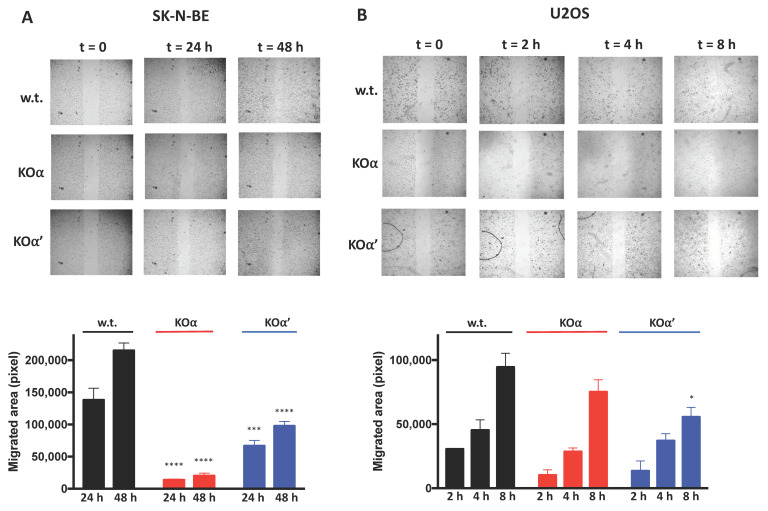
Migration of w.t. and KO clones of (**A**) SK-N-BE (KOα clone 1, KOα’ clone 1) and (**B**) U2OS (KOα clone 1, KOα’ clone 2) cells. Cell migration was assessed by wound-healing assay. Images were taken at the indicated times after the removal of the insert (t = 0). The experiments were performed three times. Representative images are shown. The bar graphs on the bottom show the quantification of the migrated areas of at least 2 different experiments, each one performed in triplicates (see Section 2 for details). Significance refers to the corresponding time in w.t. cells. (*) *p* < 0.05, (***) *p* < 0.001, (****) *p* < 0.0001.

We then evaluated the migration of cells deprived of one of the CK2 catalytic subunits by performing wound healing experiments. We noticed a slower migration of SK-N-BE w.t. compared to U2OS w.t. cells, thus the duration of the experiments was necessarily different for the two cell lines. In SK-N-BE cells, a significant closure of the gap area was observed after 48 h for w.t. cells, while in KOα’, and especially KOα cells, the closure was slower (Figure 5A). In U2OS cells, the almost complete closure was reached after 8 h for w.t. cells, while at that time the wound was still open in KO clones, especially in α’ KO cells (Figure 5B). These results suggest that both α and α’ contribute to the migration, with a more marked effect of α deletion in SK-N-BE and α’ in U2OS cells, consistent with the relative expression of each catalytic subunit in the two cell lines.

### 3.3. Effect of CK2 Targeting on Hypoxic Response and Glycolysis Enzymes

It has been reported that the inhibition of CK2 reduces stability and/or activity of HIF-1α, one of the hypoxia sensor subunits of HIF-1 [33,34,35]. HIF-1 is a transcription factor of special relevance in the metabolic rewiring of cancer cells, as it mediates cellular adaptation to hypoxic stress by upregulating several downstream targets, among which hexokinase-2 (HK2) and lactate dehydrogenase-A (LDH-A) [36], that increase glucose utilization and boost aerobic glycolysis [37]. Therefore, we decided to evaluate HIF-1α levels in our model cells.

**Figure 6:**
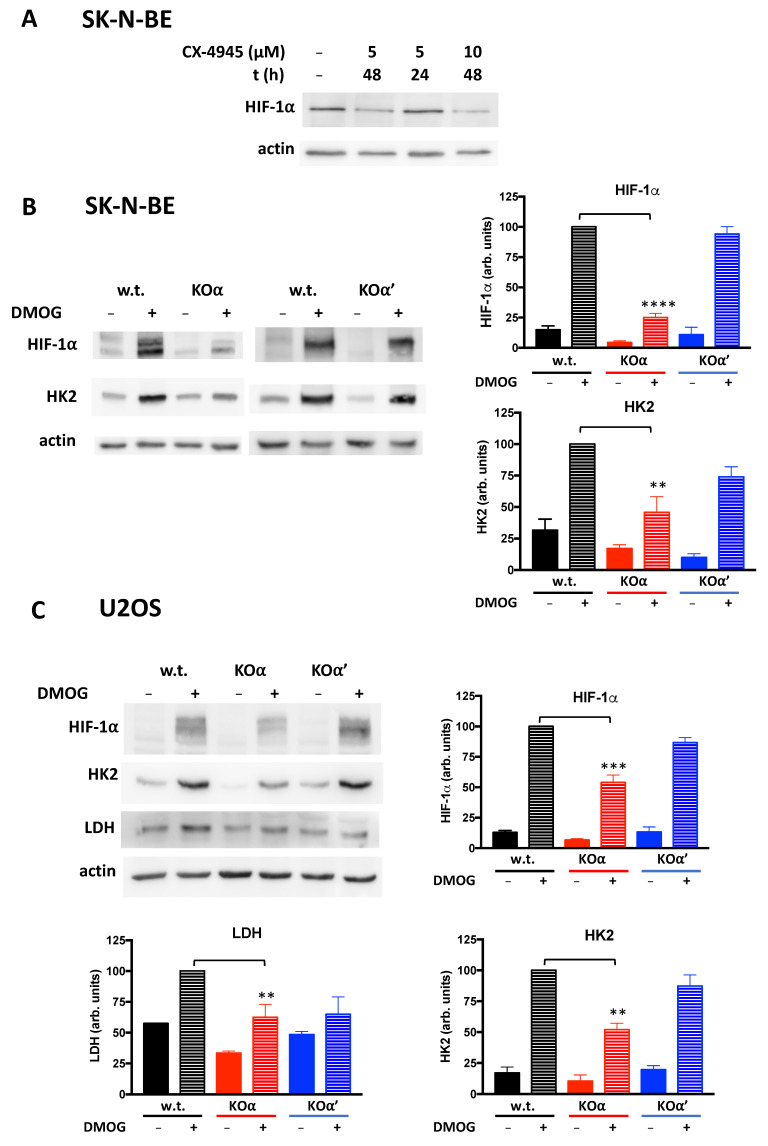
HIF-1α levels in w.t. and KO clones of SK-N-BE and U2OS cells. (**A**) w.t. SK-N-BE cells were treated with CX-4945 at the indicated time and concentration conditions. 30 μg proteins from lysates were analyzed by WB for the HIF-1α levels. Actin was used as loading control. (**B**) SK-N-BE cells (w.t or KOα clone 1, or KOα’ clone 1) or (**C**) U2OS cells (w.t or KOα clone 1 or KOα’ clone 2) were treated, where indicate (+), with 2mM dimethyloxallyl glycine (DMOG) for 20 h. 30 μg proteins from cell lysates were analyzed by WB for the indicated proteins. Actin was used as loading control. At least three independent experiments (also with different clones) were performed. Representative WB are shown. The bar graphs report quantification of HIF-1α, HK2 and LDH, as normalized mean values ± SEM of all experiments, in arbitrary units, obtained by analyses with Kodak 1D Image software. Significance refers to the corresponding condition in w.t. cells. (**) *p* < 0.01, (***) *p* < 0.001, (****) *p* < 0.0001.

As found in other cells [33,34,35], HIF-1α was reduced in response to treatment with the CK2 inhibitor CX-4945, provided that the treatment lasted enough (48h, Figure 6A). In SK-N-BE and U2OS cells, HIF-1α level was hardly detectable under normoxic conditions, but it was significantly increased by cell treatment with Dimethyloxallyl Glycine (DMOG) which induces chemical hypoxia [38] (Figure 6B,C). We observed that in both cell lines, in normoxia and especially upon hypoxia induction, the HIF-1α level was significantly lower in KOα, but not in KOα’ cells, compared to w.t. cells (Figure 6B,C). Moreover, the amount of HK2 and LDH-A induced by treatment of U2OS cells with DMOG was also lower in KOα cells, while was not significantly affected in KOα’. Similarly, in SK-N-BE the induction of HK2 by DMOG was reduced in KOα (compared to w.t. cells) much more than in KOα’ cells; in our hands, LDH-A was not induced in w.t. SK-N-BE cells by DMOG treatment, thus preventing any comparison with the KO clones (not shown).

The results described so far suggest that in tumor cells the glucose metabolism might be regulated by a single CK2 catalytic subunit. We therefore extended our study by characterizing the glycolytic phenotype of our KO clones.

### 3.4. Extracellular Lactate Production

**Figure 7:**
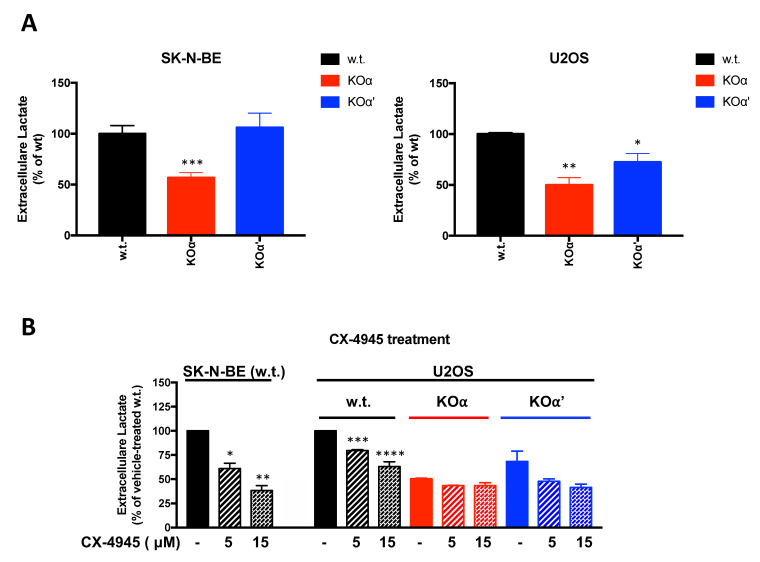
Lactate production in response to CK2 knock out or pharmacological inhibition. (**A**) The extracellular lactate secreted in the culture medium in 8 h by the indicated cells was analyzed by means of a luminescence assay. Values for each KO are the means ± SEM obtained with the different clones of the same KO. At least three independent experiments were performed, in duplicate. Significance refers to w.t. cells (**B**) Cells were analyzed for the extracellular lactate secreted in the culture medium in 6 h, in the absence or in the presence of the indicated concentrations of CX-4945. Three independent experiments were performed, in duplicate. Results (means ± SEM) are reported as % compared to the respective w.t. cells, in the absence of CX-4945. Significance refers to untreated control. (*) *p* < 0.05, (**) *p* < 0.01, (***) *p* < 0.001, (****) *p* < 0.0001.

The results shown in Figure 6 prompted us to evaluate the extracellular lactate production, as an indication of the glycolytic flux. We found that the lactate production was significantly lower in KOα clones compared to the respective w.t. cells, for both SK-N-BE and U2OS lines (Figure 7A). The deletion of α’ was instead less effective in affecting lactate secretion. As previously reported in bladder cancer cells [14], we have also found that pharmacological CK2 inhibition by CX-4945 cell treatment was able to reduce lactate production in a dose-dependent manner; consistently with a major role of CK2 α isoform, the KOα cells were less sensitive to CX-4945 treatment (Figure 7B).

These results suggest that CK2 signaling intervenes in the metabolic shift of tumor cell to glycolysis, with a more marked function of the α isoform.

### 3.5. Analysis of the Extracellular Acidification Rate

**Figure 8:**
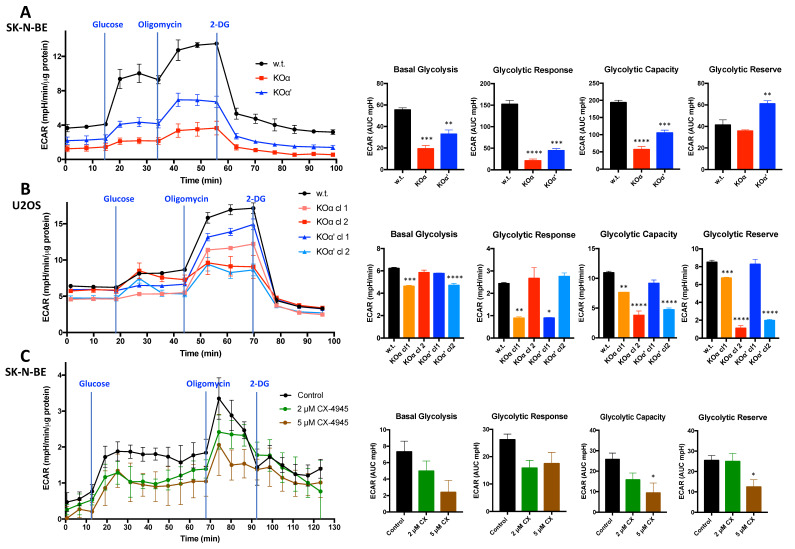
Seahorse glycolytic stress test of w.t. and KO clones of SK-N-BE and U2OS cells. The glycolysis stress tests were performed using a Seahorse extracellular flux analyzer. ECAR (extracellular acidification rate) was monitored following the indicated additions and normalized to protein concentrations. Each experiment was performed at least in quadruplicate. Values are expressed as the mean ± SEM. At least two independent experiments were performed; representative profiles are shown. (**A**) SK-N-BE cells glycolytic stress test profile (left) (KOα clone 1, KOα’ clone 2) and quantification of the experiment (right), performed with the XF software. ECAR values are expressed in AUC (Area Under Curves) units, quantifying the area of the curves corresponding to the different phases of the experiment (see Section 2). Significance refers to w.t. cells; (**B**) U2OS cells glycolytic stress test profile (left) (w.t. cells or the indicated clones) and quantification of the experiment (right), performed with the Wave software. ECAR values are expressed in mpH/min, as detected at the last measure point of each phase of the experiment (see Section 2). Significance refers to w.t. cells; (**C**) SK-N-BE cells glycolytic stress test profile, upon treatment with vehicle (control) or the indicated concentration of CX-4945 for 5 h before running the Seahorse experiment (left). Quantification of the experiment is shown on the right, performed with the XF software. ECAR values are expressed in AUC (Area Under Curves) units, quantifying the area of the curves under three points of each phases of the experiment (see Section 2). Significance refers to vehicle-treated control cells. (*) *p* < 0.05, (**) *p* < 0.01, and (***) *p* < 0.001, (****) *p* < 0.0001.

To better define the metabolic phenotype of our tumor cells, we exploited the Seahorse metabolic analyzer and performed glycolysis stress test experiments which allow to follow in real time the extra-cellular acidification rate (ECAR) caused by glycolytic activity. We found that both the glycolytic response (measured as the ECAR increase caused by glucose administration), and the maximal glycolytic capacity (assessed by inhibiting oxidative phosphorylation in the presence of glucose) were lower in SK-N-BE KO clones of either α or α’, with the strongest effect observed in KOα cells (Figure 8A). Similarly, in U2OS cells a general less glycolytic phenotype of KO clones compared to w.t. cells was observed (Figure 8B); in this case, however, there were important differences between clones. The glycolytic capacity and reserve were significantly reduced in both the two KOα clones (with also basal glycolysis and glycolytic response reduced in one of them). Instead, one of the KOα’ clones was quite similar to the w.t. cells, while the other significantly differed from them, therefore, we cannot unambiguously evaluate the real importance of this subunit in this kind of analysis. In general, we can conclude that for both SK-N-BE and U2OS cells, CK2 α seems to contribute more than CK2α’ to the glycolytic profile. As shown in Figure 8C, the Seahorse experiments performed with cells treated with the CK2 inhibitor CX-4945 demonstrated that also CK2 chemical targeting reduces the acidification rate due to glucose metabolism.

## 4. Discussion

In this work, we analyze the effect of CK2 targeting on the metabolic phenotype of cancer cells, by means of model cell lines and selective deletion of a single CK2 catalytic subunit.

Although the role of CK2 in the metabolic rewiring of cancer cells has been partly anticipated [14,15,16], our study is the first direct comparison of the specific contribution of each subunit. We found strong evidences supporting the CK2 involvement in the glycolytic phenotype of tumor cells, and suggesting a general more pronounced effect of α than α’ on this typical metabolic rewiring. This was evident, for example, on HIF-1α amount and activation, and consequent HK2 and LDH levels (Figure 6), on lactate production (Figure 7), on Seahorse glycolytic profile (Figure 8) (although with some variance among different clones, whose explanation will require further investigation). On these features, for both cell lines we observed a stronger divergence of KOα than KOα’ from w.t. cells. In the case of KOα’ U2OS cells, the lactate production was even equal to that measured in w.t. cells (Figure 7A). This is in partial discrepancy with the Seahorse experiments of Figure 8A (where one KOα’ clone is less glycolytic than w.t. cells). However, it has to be reasoned that the lactate assay provides a picture of a static situation at a certain time, while the Seahorse experiment is a real-time analysis, ideally measuring the acidification rate due not only to lactate production. In any case, both approaches support a major role for CK2α.

Our observations on the isoform-specific effects do not seem to depend on the amount of residual CK2 catalytic activity, which is different in the two cell lines (see Figure 1), and, instead, correlates with the migration potential (Figure 5). In contrast, specific functions of α respect to α’ appear to play a role in the regulation of metabolic rewiring. CK2 isoform-specific substrates/partners/functions have been already reported [11,31,39,40,41]; further studies will be necessary to assess their effective implication in cancer cell metabolism and to identify novel targets and mechanisms that might be implicated. However, it is also important to notice that, as previously observed in other cell lines [31], the KOα clones display a reduction of the β CK2 regulatory subunit, which is not (or only marginally) appreciable in KOα’ cells (Figure 1). Therefore, it is likely that KOα cells have a lower cellular amount of the tetrameric CK2 holoenzyme. This observation further complicates the interpretation of our data, since it has been shown that knocking out β is detrimental to the phosphorylation of a number of CK2 targets but not of others [42]. In fact, monomeric and tetrameric CK2 display different activity towards a panel of selected substrates [7]. We cannot exclude, therefore, that the more evident effects observed in KOα cells are due to the lower amount of tetrameric CK2. In discriminating between the importance of CK2α and tetrameric CK2, an advantage would potentially derive from the establishment of KOβ cells; however, for the time being, any attempt to produce tumor cell devoid of β has failed (unpublished results). Implicitly, this suggests that the tetrameric form of CK2 is essential for cancer cell survival.

Several inhibitors are available for CK2 (with two of them, CX-4945 [43] and CIGB-300 [44] already at a clinical level). Tumor cells have been widely documented to respond to CK2 inhibitors, alone or in combination with conventional drugs (as reviewed in [45]). Concerning the cell lines used in this work, Figure S1A shows that SK-N-BE cells are sensitive to the CK2 inhibitors CX-4945, TDB (Tetra-bromo-deoxyribofuranosyl-benzimidazole) [46] and K137 (*N*′-(4,5,6,7-tetrabromo-1Hbenzimidazol-2-yl)propane-1,3-diamine, also known as 2b) [47,48], with IC50 in the μM range (see Figure S1A). As for U2OS cells, we have already published that CX-4945 reduces their viability, with an IC_50_ around 4 μM [23]. Interestingly, we have also preliminary evidence suggesting that the combined treatment with a CK2 inhibitor (CX-4945) and a metabolic inhibitor (2-DG) produces synergistic effects on U2OS cell viability (Figure S1B), allowing to reduce the effective doses of each drug. The calculated combination index of 0.37 supports a synergistic effect. These results give a particular significance to the work presented here, in the perspective of future therapeutic strategies based on CK2 targeting. 

Most CK2 inhibitors are equally effective towards the different CK2 forms, but attempts have been done to develop strategies/compounds able to discriminate between them, either tetrameric vs monomeric [49], or α vs α’ [50]. The knowledge of the mechanism(s) underlying the contribution of each CK2 form to cancer metabolic phenotype is crucial for the choice of the correct inhibition strategy for therapeutic applications. 

Apart from the α/α’ issue, our results unequivocally support the CK2 implication in the metabolic glycolytic phenotype of different tumor cells, and suggest that CK2 inhibition may hamper the energy supply strategies of cancer cells, thus offering further opportunities of therapeutic plans.

## Data Availability

Data presented in this study are contained within this article and the supplementary materials, or available upon request to the corresponding author.

## Supplementary Materials

The following are available online at https://www.mdpi.com/2073-4409/10/1/181/s1, Figure S1: Effect of CK2 inhibitors on cell viability.
