# Supplementary material for: Contribution of the CK2 Catalytic Isoforms α and α’ to the Glycolytic Phenotype of Tumor Cells
Source: Cells. 2021 Jan 18;10(1):181. doi: 10.3390/cells10010181 (PMC7831337; doi:10.3390/cells10010181)
Supplement: Supplementary file 1 [file cells-10-00181-s001.pdf]

A

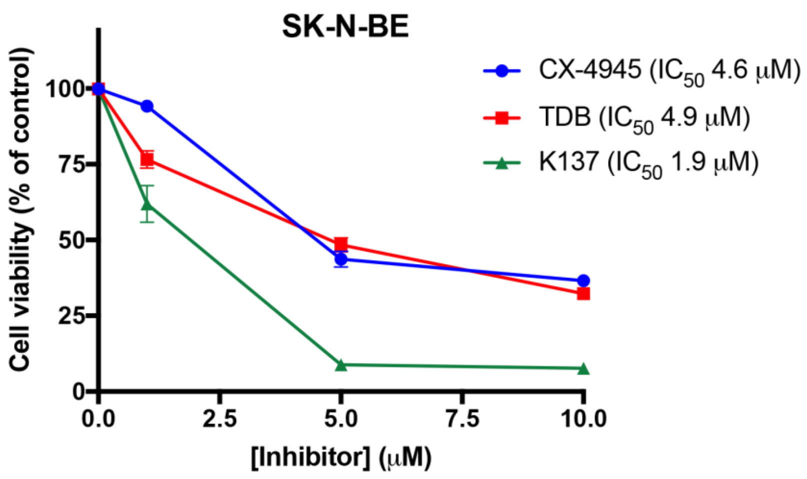

B

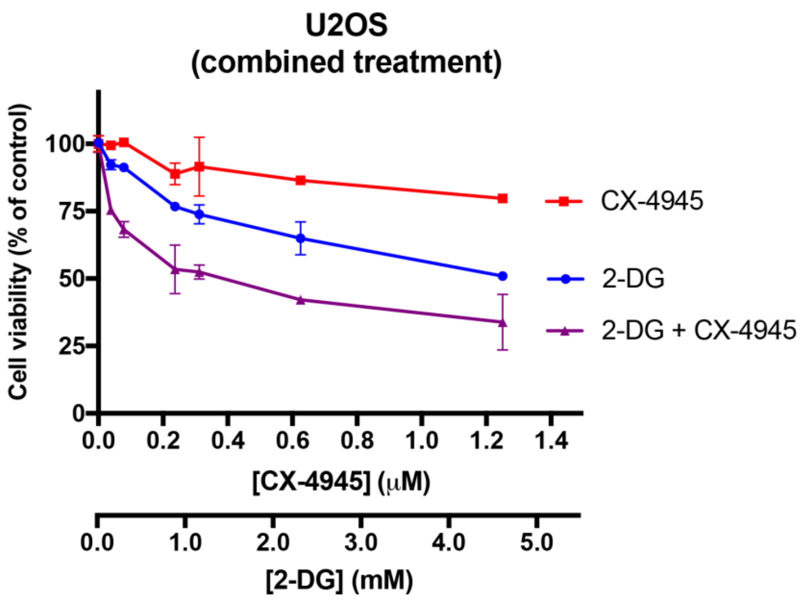

	IC_{50} CX-4945 (μM)	IC_{50} 2-DG (mM)
**Single treatment**	> 1.25	> 5.0
**Combined treatment**	0.386	1.54

Figure S1. Effect of CK2 inhibitors on cell viability. Cell viability, measured by the MTT method, is shown as percentage of vehicle-treated cells (control). The IC_{50} values (concentrations reducing cell viability to 50%) were calculated with the GraphPad Prism 7 software. (**A**) SK-N-BE cells were treated for 48h with increasing concentrations of the CK2 inhibitors CX-4945, TDB, or K137. The IC_{50} values are reported on the side of each inhibitor (**B**) U2OS cells were treated for 24 h with increasing concentrations of CX-4945 (upper X-axis), or 2-DG (lower X-axis), or both compounds in the fixed ratio of 1:4000 (CX-4945:2-DG). The table in the bottom reports the IC_{50}values for the two compounds, either alone or in combined treatment.
